# Precision medicine for COVID-19: a call for better clinical trials

**DOI:** 10.1186/s13054-020-03002-5

**Published:** 2020-06-02

**Authors:** Gentle Sunder Shrestha, Hem Raj Paneru, Jean-Louis Vincent

**Affiliations:** 1 Department of Anaesthesiology, Tribhuvan University Teaching Hospital, Maharajgunj, Kathmandu, Nepal; 2 Department of Intensive Care, Erasme University Hospital, Université Libre de Bruxelles, Route de Lennik 808, 1070 Brussels, Belgium

The present coronavirus disease (COVID-19) has emerged as a global pandemic, infecting millions and killing thousands of patients. Even though many questions remain unanswered, knowledge on the pathophysiology of COVID-19 has improved substantially around many aspects, including the development of the virus, the role of ACE2 receptors, the type and severity of organ involvement, the importance of coagulopathy and endotheliopathy, and the role of disproportionate cytokine response [1]. There is growing evidence that patients with COVID-19 are in fact having a heterogeneous disease, going well beyond the differences in lung compliance and ventilation-to-perfusion mismatch [2]. Elucidating the variable phenotypes and the underlying pathophysiology can be a key step to individualize therapy.

## Available therapies

Treatment of COVID-19 is largely supportive as yet there is no effective antiviral drug, vaccine, or antibody against the virus. Hundreds of trials are underway globally, examining the efficacy of different drugs, convalescent plasma, herbal medicines, etc. (search on trials.gov reveals 609 trials registered till 20 April 2020). The pharmacological agents that have been investigated include various agents like camostat mesylate, tocilizumab and sarilumab, arbidol, chloroquine and hydroxychloroquine, lopinavir/ritonavir, darunavir, ribavirin, remdesivir, favipiravir, interferon alpha, famotidine, etc. The agents have variable mechanism of actions, ranging from inhibition of entry of viral particles into the cells and inhibition of various enzymes associated with viral replication to an improvement of the host immune response to the virus [3].

## Trial design

As the pandemic is rapidly spreading, clinicians treating COVID-19 are in desperate need of an effective therapy, as development of an effective vaccine remains a remote possibility. In the face of the pandemic, conducting well-designed clinical trials aiming to explore effective therapeutic options is associated with both challenges and opportunities.

A large number of negative trials are a major problem in critical care medicine [4]. This is largely due to the heterogeneous patient population, with different biological mechanisms and different biological responses to a disease in individual patients. Rather than conducting trials using the conventional trial designs and poor patient selection, precision-guided studies have a greater potential to yield positive results. In the face of the global pandemic, we should minimize the risks of negative trials. The longer time lag to explore effective therapy will be associated with larger loss of health and lives, in addition to waste of resources and scientific efforts.

**Fig. 1:**
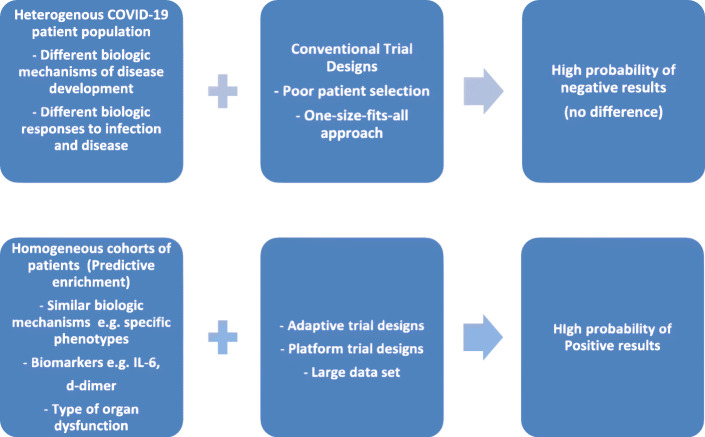
Precision medicine for better clinical trial design in COVID-19

Better selection of more homogeneous groups of patients can be feasible in COVID-19 compared to other critical illnesses because of the large number of patients needing hospitalization [5]. Incorporating predictive enrichment strategies can help to identify and thus target specific phenotypes, potentially raising the possibility of positive trial outcomes (Fig. 1) [6]. We should precisely choose therapies according to the specific needs of individual patients and not offer the same treatments for all. Enrichment of studies can be done by selection of patients with a strong likelihood of a response to an intervention, thereby reducing study noise, sample size, and study-associated harm. Homogeneity in selecting patients can be guided not only by advanced molecular testing for biomarkers but also by the simple tools like clinical features of patients including clinical stage of disease, vital signs measurement, laboratory data, ventilator settings, and other types of organ dysfunction.

Some examples of precision medicine-guided trial designs may be, and not limited to:

- Trial of hydroxychloroquine in individuals at high risk of acquiring infection, but not yet infected, those without cardiovascular disease, with normal QTc, avoiding concurrent medications that can prolong QT interval, cautiously selected dosing, dose adjusted to renal function, avoiding and rapidly correcting dyslectrolytemia [3, 7].
- Trial of anti-inflammatory agents like corticosteroids or anti-cytokine agents like tocilizumab in patients with or at risk of endothelial dysfunction or endotheliitis or in patients with an elevated level of interleukin-6 [3, 8].
- Trial of anticoagulation in patients with sepsis-induced coagulopathy or marked elevation of d-dimer and without contraindication for anticoagulation [1, 9].

The data pool can be increased by enrolling multiple centers in the trials rather than conducting multiple small trials at individual centers. Large multicenter trials can also have financial, political, and academic gains [10]. As we have a large number of patients that may meet eligibility criteria for individualized therapies, we have the potential of getting large cohorts of patients in a shorter time period. A robust data infrastructure developed by combined efforts of clinicians, researchers, and data scientists is the need of time. Adaptive trial designs that quickly discontinue poorly performing therapies and involve international, high-enrolling RCTs that facilitate “1-stop shopping” at the point of care for the evaluation of different therapies are an attractive and appropriate option at the present context [11].

There are too many small trials with limited chances of therapeutic success. We urge the scientific community to unite and meticulously design trials fostered by the concept of precision.

## Data Availability

Not applicable.

## Abbreviations

COVID-19: Coronavirus disease 19
ACE2: Angiotensin-converting enzyme 2
QTc: Corrected QT
RCT: Randomized controlled trial
